# A Pilot Study to Validate a Wearable Inertial Sensor for Gait Assessment in Older Adults with Falls

**DOI:** 10.3390/s21134334

**Published:** 2021-06-24

**Authors:** Guillermo García-Villamil, Marta Neira-Álvarez, Elisabet Huertas-Hoyas, Antonio Ramón-Jiménez, Cristina Rodríguez-Sánchez

**Affiliations:** 1 Centre for Automation and Robotics, UPM-CSIC, 28500 Madrid, Spain; guillermo.gv@csic.es (G.G.-V.); antonio.jimenez@csic.es (A.R.-J.); 2 Department of Geriatrics, Foundation for Research and Biomedical Innovation of the Infanta Sofía University Hospital and Henares University Hospital, (FIIB HUIS HHEN), European University, 28702 Madrid, Spain; marta.neira@salud.madrid.org; 3 Physical Therapy, Occupational Therapy, Rehabilitation and Physical Medicine Department, Rey Juan Carlos University, 28922 Madrid, Spain; 4 School of Experimental Sciences and Technology, Rey Juan Carlos University, 28933 Madrid, Spain; cristina.rodriguez.sanchez@urjc.es

**Keywords:** frailty, gait analysis, IMU, mobile app, telemedicine, pedestrian dead reckoning, assistant

## Abstract

The high prevalence of falls and the enormous impact they have on the elderly population is a cause for concern. We aimed to develop a walking-monitor gait pattern (G-STRIDE) for older adults based on a 6-axis inertial measurement (IMU) with the application of pedestrian dead reckoning algorithms and tested its structural and clinical validity. A cross-sectional case–control study was conducted with 21 participants (11 fallers and 10 non-fallers). We measured gait using an IMU attached to the foot while participants walked around different grounds (indoor flooring, outdoor floor, asphalt, etc.). The G-STRIDE consisted of a portable inertial device that monitored the gait pattern and a mobile app for telematic clinical analysis. G-STRIDE made it possible to measure gait parameters under normal living conditions when walking without assessing the patient in the outpatient clinic. Moreover, we verified concurrent validity with convectional outcome measures using intraclass correlation coefficients (ICCs) and analyzed the differences between participants. G-STRIDE showed high estimation accuracy for the walking speed of the elderly and good concurrent validity compared to conventional measures (ICC = 0.69; *p* < 0.000). In conclusion, the developed inertial-based G-STRIDE can accurately classify older people with risk to fall with a significance as high as using traditional but more subjective clinical methods (gait speed, Timed Up and Go Test).

## 1. Introduction

Falls are one of the most significant clinical problems in the elderly population. One-third of the elderly living in the community falls every year, and about half of them suffer repeated falls. Additionally, 1 in 10 elderly people with falls will be hospitalized due to the falls, and only half of them will live a year [1,2]. Therefore, the evaluation of patients at risk of falling is key to taking preventive measures to avoid its prevalence.

The assessment of risk of falling can be implemented by analyzing its gait and balance, and daily activities [3,4]. Gait speed and step lengths are detected as the main parameters for identifying people with high fall risk [5,6]. In primary and specialized care, this can be achieve using inexpensive methods based on physical examination and functional tests such as the gait speed test, the Short Physical Performance Battery (SPPB) [7], and the Timed Up and Go Test (TUG) [8], and the Environment-Adaptive TUG (EATUG) fall risk assessment system [9], which is an improvement of the standard TUG test. These conventional functional tests provide information on running times and the qualitative aspects of gait. However, they are, to some extent, subjective by using verbal instructions to the patient, manual temporal measures (e.g., handheld stopwatches) and they do not provide information on many other gait parameters of interest for recognizing aspects related to falls, such as cadence, stride length or swing phase time, for example.

The analysis of gait can be obtained using more objective and accurate instruments. Some of the commercial tools, that can be used for risk of falling assessment (and other applications), are: (1) optical cameras (e.g., Vicon or OptiTrack), and (2) pressure pathways (e.g., Gaitrite [10,11]). They provide precise information on gait pattern, step speed, step length & width, and static and dynamic balance behavior under controlled conditions. However, the use of these tools is still not widespread; it is practically limited to monographic units with gait laboratories (i.e., specialized hospital environments) or in the field of motion analysis research. This is because of the high cost of this kind of equipment and the time and space required to do such detailed analyses. Therefore, a technology able to estimate with accuracy those gait parameters but with less-infrastructure and much lower costs is needed: inertial sensing is that disruptive technology.

The use of inertial sensors has emerged as a technology useful in different gait analysis studies [12,13,14,15,16,17,18]. The main studies and innovations found in the literature about inertial sensing have already been reviewed in several surveys [19,20,21]. An inertial sensor is a portable, low-cost, lightweight device called inertial measurement unit (IMU). A IMU can be placed at different locations on the body, especially on the leg, back, ankle, and foot, to extract motion gait parameters. Inertial sensors measure raw accelerations and turn rates by orthogonally oriented accelerometers and gyroscopes. This raw data must be post-processed to obtain gait parameters with reasonable accuracy.

Gait parameters can be estimated from IMUs via three types of inertial estimation algorithms: (1) human gait models, (2) abstraction models (artificial intelligence or regression) and (3) direct inertial integration (or INS) [22,23,24]. The first method requires defining the kinematic human gait model, using known parameters such as leg length and walking style. The IMU can measure joint rotations or heap oscillations and deduce the cadence and parameters such as speed of stride length. This estimation depends on the particular subject and must be calibrated, which is a problem when we want to study a large group of subjects. In the second approach (abstraction models), the problem is considered as a black box, where a set of features are extracted from the IMU, but also social-demographic features of the subject under study. This kind of method uses artificial intelligence algorithms, such as neural networks, or regressions models, in order to try to learn gait parameters in a supervised way (a large database of inertial log files and known gait parameters given as examples). This approach has a main drawback: the need of new trainings (learning or model fitting) with new databases each time the population changes, apart from a limited accuracy (higher than 5%).

In the last, and third, approach (INS), a direct double integration of acceleration and angular rates is used. This approach requires sophisticated algorithms to eliminate the noise typical of microelectromechanical system (MEMS)-type IMUs, which, after double integration, cause significant drifts in spatial and angular estimations. To minimize this effect, zero-update velocity (ZUPT) [25] during stance is performed, which force the use of the IMU on the foot of the subject [3]. Apart from these challenges and foot-location restriction, this method has a significant advantage. It does not need to use any human model, no calibration of the user features (e.g., leg-length), and no learning process must be performed. The estimation of gait parameters can be valid for different kind of users (young or elder, with or without pathologies, moving fast or slow). A few studies perform a direct INS integration-based walking speed estimation algorithm [13,26,27] with IMUs attached to feet with a reported accuracy of about 3%.

The technology used for gait analysis is not the only aspect that matters; the evaluation site and conditions have an important influence on the assessment. For example, even for all of the abovementioned technological solutions (i.e., conventional, complementary instruments, and inertial devices), examinations by a doctor require long consultation times and relatively wide indoor spaces, the patients tend to outperform, and the test surface is quite ideal. Additionally, the execution of these tests requires the understanding and collaboration of patients, which is difficult in those with cognitive impairment. Under these circumstances, it would be desirable to perform tests with portable devices (such as IMUs) but in “real word” conditions. This assessment could be done at home or in the patient’s everyday street environment with various terrain conditions (slopes, sidewalks, steps, etc.) and where falls usually occur. Conducting more extended walking distance tests (more than half an hour), and without the supervision of a doctor, patients do not tend to outperform, which is good for reliable gait analysis and risk of fall assessment, or in other pathologies or syndromes such as frailty.

In this paper, we aimed to develop an accurate INS-based inertial solution (G-STRIDE), easy to use by patients and clinical stuff, and with special focus on testing the structural and clinical validation of old patients. For that purpose, we propose to address the following innovations.

Firstly, we propose and present the development of an inertial INS-based device (the G-STRIDE device) that allows gait assessment without needing any learning or calibration phase (valid for any kind of persons independently of its age). An approach with high accuracy, and a potential for a larger range of gait parameter estimations (e.g., stride length, speed, cadence, rotation angles, clearance, etc.) when compared with model-based algorithms.

Secondly, our proposal includes the management of the processed gait information through a mobile application (the G-STRIDE App) to make it accessible to clinicians (easy to use and pre-classification of risks indicators, or pre-frail or frail categorization).

Thirdly, we evaluate differences between people with and without falls using classic functional procedures and inertial device parameters (the G-STRIDE Assessment). As falls in older adults are deeply linked to frailty status as it is one of the main pathogenic factors, we are interested to explore the relationship between gait parameters and frailty status in older adults and studying possible cut-off points to classify patients according to device parameters.

This paper is organized as follows: Section 2 describes the pilot study for fall assessment, the design and implementation of the G-STRIDE solution, including sensors, hardware, communication, data processing, and user interface, as well as the statistical analysis. The relevant results from the clinical point of view, in gait analysis and groups of patients, and the validation of the technology for frail detection, are presented in Section 3. Finally, a global discussion and some conclusions are shown in last two sections.

## 2. Materials and Methods

### 2.1. Design of Pilot Study

A pilot observational case–control study was conducted in the Community of Madrid. The study was approved by the Research Ethics Committee of the Hospital Universitario de la Paz (Registration No: PI-4486), following the ethical principles for medical research involving human subjects of the Declaration of Helsinki. All participants signed the informed consent for participation in the study.

### 2.2. Sample

All the participants were adults from the Falls Outpatient Clinic in the Geriatric Department of the Hospital Universitario Infanta Sofía (Madrid, Spain) included using non-probabilistic consecutive sampling. The inclusion criteria are a modification of the criteria for referrals to specific units for the evaluation and treatment of falls in the elderly, proposed by the American Geriatrics Society (AGS) and the British Geriatrics Society (BGS) [28]: participants over 70 years of age who could walk without assistance from another person and met one of the three criteria: (a) One fall or two or more falls with consequences in the last year; (b) gait and balance disorder or fear of falling; (c) post-fall syndrome. Exclusion criteria were having a terminal illness with a life expectancy of fewer than six months or not providing informed consent to participate.

The participants’ assessment was carried out during a single visit to the Falls Out-patient Clinic and usual assessment was conducted to collect the study variables described in Falls Assessment. Finally, the device was placed in the shoe, and the participant was asked to walk freely for 30 min (recommended walking time for older people based on scientific evidence [29]). At the end of this time, the participant returned to the out-patient clinic to give back the device.

### 2.3. Falls Assessment

Falls assessment was done according to clinical practice of geriatricians in Out-patient Clinics and included:

- Age, sex, height, weight, height, and body mass index (BMI).
- Global cognition measured using Reisberg Global Deterioration Scale (GDS) [30].
- Self-report physical activity: it is assessed according to frailty criteria considering sedentary if they walk less than 3 h per week in the case of men or less than 1 h per week in women.
- Assessment of frailty status using the Standardized Frailty Criteria (SFC) that includes five criteria: weight loss; measured weakness; self-report exhaustion; measured slowness; low activity questionnaire. The score range 0 to 5 being Frail when ≥3 criteria are present, Pre-frail when 1 or 2 criteria are present and Robust or Non-frail when there are no criteria present [31].
- Short Falls Efficacy Scale-International (Short FES-I): This scale measures “fear of falling” or, more properly, “concerns about falling” in older adults living in the community. The scale includes different activities of daily living and it scores from 7 to 28 seven (no concern) to 28 (severe concern). From 9 points onwards, it is considered moderate concern [32].

Functional Tests:

- Four-Meter Gait Speed (FMGS): patients are instructed to walk 4 m at their “*usual speed*”. Timing with a stopwatch began when the first foot pass the starting line and ends when the same foot pass the finish line. Three-time trials are taken, choosing the best of them. Gait speed below <0.8 m/s suggests an increased risk of frailty and below 0.6 m/s an increased risk of disability and they both need further clinical review [33].
- Short Physical Performance Battery (SPPB): is an objective assessment tool for evaluating lower extremity functioning in older persons combining three tests: gait speed, chair stand and balance tests. It has been used as a predictive tool for possible adverse events and disability. The scores range from 0 to 12 with higher scores indicating better lower body function.

In order to classify participants as frail, pre-frail and non-frail or robust, the following cut-offs were used: SPPB < 4 (with disability), SPPB 4–6 (frail), SPPB 7–9 (pre-frail), SPPB 10–12 (non-frail or robust) [7].

- Timed Up and Go Test (TUG): The patient is observed and timed while he rises from an armchair, walks 3 m, turns, walks back, and sits down again. A score of ≥14 s has been shown to indicate high risk of falls [34].

### 2.4. Designed G-STRIDE Architecture

**Figure 1:**
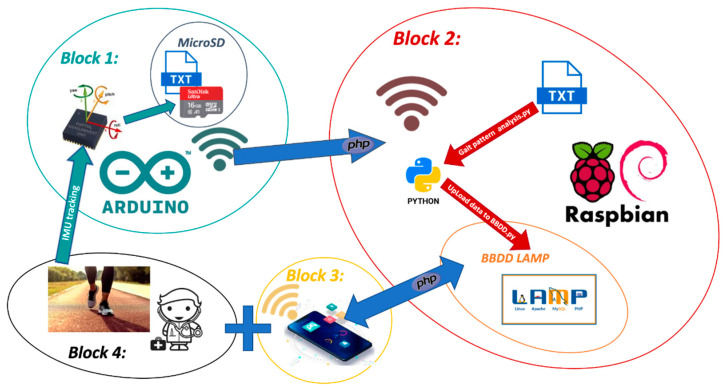
Architecture of G-STRIDE solution.

Figure 1 shows the architecture of the G-STRIDE solution and its component blocks:

#### 2.4.1. Block 1: Inertial Device

The inertial device includes a sensor, the sampling of accelerations and turn rates, low-level signal processing, and wireless communications. The 6-axis IMU’s raw signals (i.e., acceleration, angular rotation, and the timestamp of the microcontroller) were recorded on a microSD card during the test, and when the test was completed, they were sent wirelessly, using WIFI communication (IEEE 802.11) to Block 2. The G-STRIDE inertial device was placed on the subject’s foot in a non-intrusive manner to continuously sample the gait pattern for 30 min during regular walking activities.

#### 2.4.2. Block 2: Cloud Processing

The cloud processing and storage block was implemented using a Raspberry-Pi consisting of a signal processing stage in Python to extract the gait pattern (stride length, cadence, speed, etc.) using INS-based algorithms [35] (also known as Pedestrian Dead Reckoning or PDR techniques). A portable server stores the generated gait information. The server was implemented in the Raspberry using a Linux, Apache, MySQL, PHP/Python (LAMP) system developed by members of the SENIALAB and LABTEL groups of the URJC [36]. This provided an added value to the system: it is a portable and low-cost system that allows a quick and straightforward installation in any gait assessment practice.

#### 2.4.3. Block 3: Management App

The management App is the interface between the user and the system. It is accessible, intuitive, and open to the inclusion of new elements. The clinician can consult each patient’s variables and analyze the data to automatically obtain the correlation with measurements of self-perception and influence on daily activities. The project’s clinical team can access the content and management of this website to analyze the data collected by the users and activate alert mechanisms or change routines/tasks.

#### 2.4.4. Block 4: Tests with Patients

A series of conventional tests (FMSG, SPPB, TUG, Short FES-I, SFC) were carried out to determine if the device could correlate with the usual scales for frailty identification. In these tests, the subjects would perform the traditional metrics. Additionally, they would carry the sample device during thirty minutes of free walking.

### 2.5. G-STRIDE Hardware and Software Implementation

**Figure 2:**
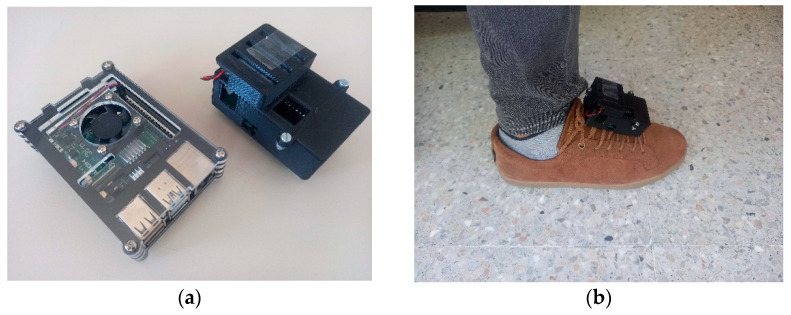
Server (Raspberry) and G-STRIDE (“sampling device”). In picture (**a**) the server implemented in a Raspberry Pi model 4B and the G-STRIDE device with its case are displayed. In picture (**b**) sampling device is attached to the shoe with laces or Velcro.

The implementation of the system consists of four parts, sampling device, data processing, data storage and data visualization. In Figure 2 sampling device attachment and server are shown (Video S1).

#### 2.5.1. Implementation of G-STRIDE Sampling Device

**Figure 3:**
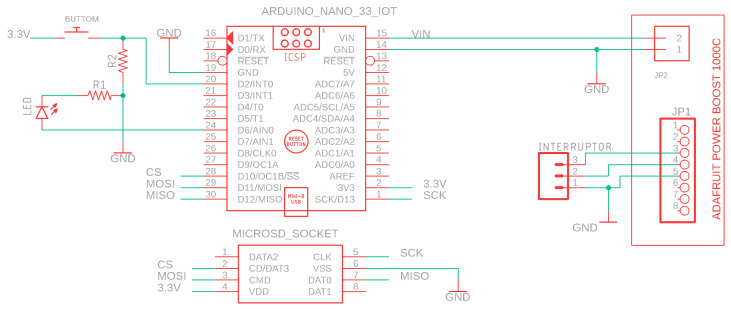
Schematics of the sampling device PCB.

This section explains the implementation of the G-STRIDE sampling device. This device was designed to take inertial measurements and generate a walking pattern in post-processing and Wi-Fi connectivity. For this reason, the Arduino NANO 33 IoT board was used (Arduino, Scarmagno, Italy), which has a 6-axis IMU and a WiFiNINA module. A PCB board was designed to incorporate the Arduino board together with a voltage booster for the LIPO battery and a microSD card reader as shown in the schematics of Figure 3.

The booster, Adafruit Power Boost 1000C (Adafruit, New York, NY, USA) was necessary to power the Arduino, as it needs a 5 V input and the LIPO battery only provides 3.7 V. The microSD card reader allowed for recording the IMU values on the microSD during the test, this data was recorded synchronously with a timestamp. When the test finished, the content was sent by Wi-Fi. The button shown in the schematics of Figure 3 allowed the user to interact with the G-STRIDE device.

Finally, the switch allowed the user to turn the G-STRIDE device on and off. The IMU used was the LSM6DS3 from the manufacturer STMicroelectronics (Geneva, Switzerland). It had an accelerometer with three orthogonally oriented axes and a gyroscope with three orthogonally oriented axes. The IMU can be adjusted in different ranges, 2/4/8/16 g for the accelerometer and 125/250/500/1000/2000 dps for the gyroscope. The IMU frequency was set to 104 Hz, this frequency was adjusted according to the technical characteristics of the IMU and the frequency used in related works [21], which is about 100 Hz.

#### 2.5.2. Data Processing

All processing of the data recorded was performed using Python on the Raspberry server. Raw IMU data, consisting of linear accelerations and angular velocities, must be integrated to obtain spatio-temporal gait parameters. The estimation of parameters like stride length, average speed, cadence, and swing time were established based on medical requirements.

For this issue, it was first necessary to detect the foot’s stance moment, i.e., to detect the moment at which each step occurs. The stance phase is detected based on local standard deviation of the acceleration magnitudes, following the methodology explained in [35]. For this purpose, events such as the heel strike are detected. The stance phase was detected by applying two conditions that must be satisfied; firstly, it is required to detect a transition between high and low accelerations with an upper threshold, and then these low accelerations must be below a lower threshold during a fixed window. It was determined that the thresholds that best met the test conditions were 1.6 m/s^2^ for the upper threshold and 0.8 m/s^2^ for the lower threshold. Moreover, it was necessary to integrate the acceleration twice, between two consecutive step detection events, and translate the sensor’s local coordinate system to the global one, updating the orientation matrix with the gyroscope values. Therefore, noise in these sensors can lead to significant errors in position estimation due to drift. Studies previously carried out by [37] reflect these effects. To reduce these errors of estimation, the ZUPT correction was implemented, which was clearly developed and explained by [35]. During the stance phase, the sensor is assumed to not be moving. Therefore, the velocity obtained during this phase was forced to 0. This error during the stance phase was used to remove it from the rest of the measurements present during the swing phase, since drift is an error that accumulates and increases over time. Then with double integration of IMU data, spatial parameters such as stride lengths can be easily estimated. Cadence was obtained with the number of steps detected divided by the time in which the subjects were walking. For walking speed, we directly used the speed estimated by ZUPT algorithm. Swing times were calculated by obtaining the difference in the time stamps of the end of a stance phase (toe off) and the moment when the foot is detected to contact the ground again (heel strike).

#### 2.5.3. Data Transfer and Data Storage

**Figure 4:**
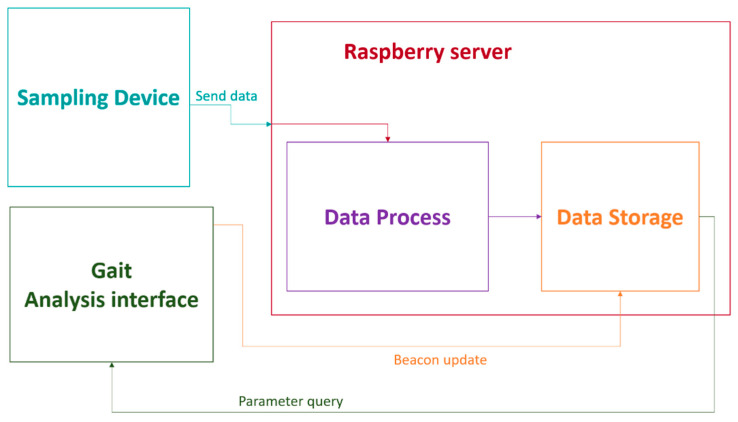
Communication and data transmission.

In Figure 4 the data transfer process, through the blocks 1, 2 and 3 of the architecture, is described. The data recorded during tests in “Block 1” (inertial or sampling device) is stored in a text document on the microSD and sent by WIFI. On the server, these data are collected using a PHP file that stores it in a text document. When all data are sent, algorithms that calculate the walking pattern parameters are executed (red arrow, Figure 3). When the gait pattern parameters are obtained, they are uploaded to the BBDD with the patient identification number corresponding to the beacon used to send the data (purple arrow in Figure 3).

Finally, the data can be consulted through a mobile application, which corresponds to “Block 3” (management App) and allows the identification number of each patient to be updated with that of each beacon, orange arrow in Figure 3. The raw sensory information recorded by Block 1 is additionally sent wirelessly to the URJC cloud server (IMB + Watson). Work is also being done on a Raspberry 4-based server that allows for duplicate storage of the nodes of the clinical units that receive patient data. Thus, if a clinical entity does not want to upload it to the cloud, it can do so in a “cloud” managed by a Raspberry 4 customized for a specific clinical entity. This allows having a record with a backup of patient data for the duration of a project. The server side has been implemented as a remote monitoring system that allows ubiquitous data access. It is a modular design that allows several databases from different sampling devices and simultaneous access to them from different consultation devices: computer, tablet, smartphone, etc. The system has been designed to allow the database to be replicated on other remote servers if necessary, adding backup capabilities to the system and allowing remote collaboration between different users and locations. Grafana v7.3.0 is used as the front-end for the visualization of the time-series data. A series of dashboards are used to monitor information in real-time. All stored data are time-stamped and associated with a specific node, allowing the comparison of results between locations. Dashboards are connected to a web page hosted on the server that allows the user to select the node to be consulted. HTML5, CSS3, and JavaScript have been used for web design.

Work is being done to evaluate the nodes’ compatibility with a server hosted on IBM Cloud. This communication has been implemented through a data frame, which contains the information coming from the sensors. This frame’s structure consists of a header with relevant information with the parameters they use for their communication protocol in the application layer. The second part consists of a message of variable length depending on the data included. A hexadecimal frame is used for this purpose. A timestamp is also included. Once uploaded to IBM Cloud, the data are sent to the external database server for remote management automatically using a Python script. MySQL Server 5.7.29 on an Ubuntu 18.04 LTS (Long Time Service) machine has been used as the server.

#### 2.5.4. Visualization Interface for Gait Parameters

**Figure 5:**
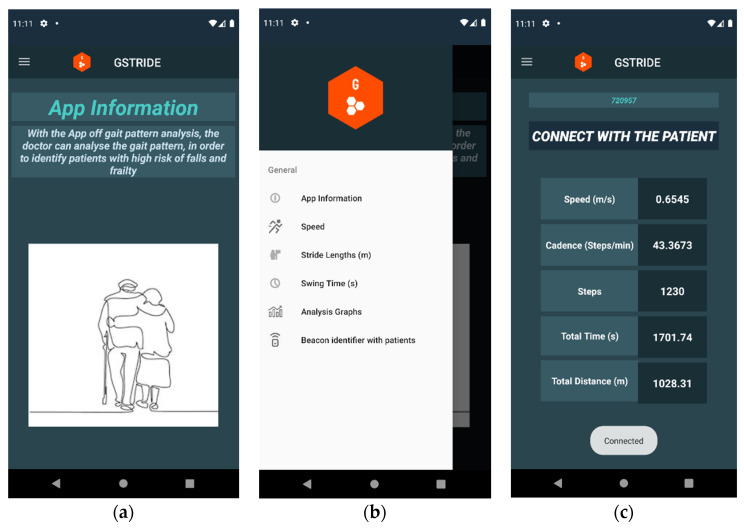
View 1: mobile application. In screenshot (**a**) entry view of the App is shown. In screenshot (**b**) a toolbar is displayed for navigation through the App. In screenshot (**c**) parameters of each subject are displayed.

**Figure 6:**
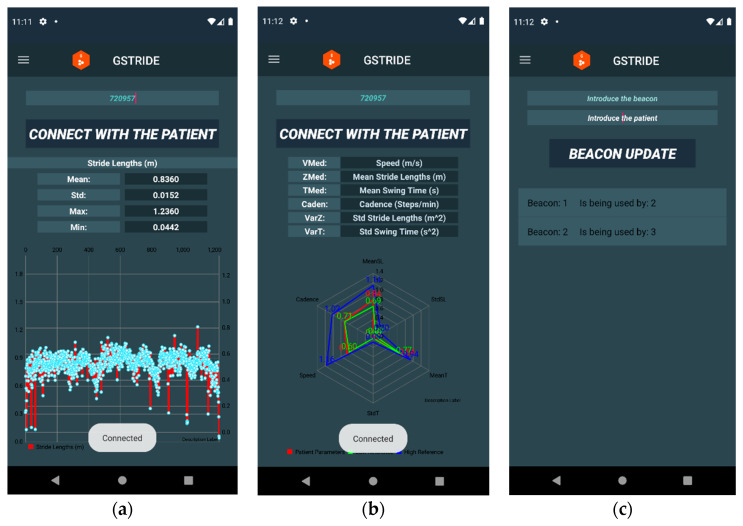
View 2: Mobile application. In Screenshot (**a**), parameters of each subject are displayed with text views and graph with each stride length of the subject during the test. In the second screenshot (**b**) a radar graph is displayed with some parameters of the selected patient (red line) and compared with top (blue line) and low (green line) reference values of each parameter of the total sample. In the third screenshot (**c**), each beacon can be updated with the subject identifier.

The mobile app for consultation and visualization of patient gait pattern data was developed in Android Studio Version 4.1.2. It contains different tabs that can be accessed from a drop-down menu, text views to monitor the parameters, and graphs to help their interpretation. The following tabs are available in the app: Application Information, Speed, Strides, Swing Times, Graphs, and Waypoint Identifier (Figure 5 and Figure 6). Besides, a tab called Devices is available, which allows the physician to update the patient’s identification using the number of the device.

### 2.6. Statistical Analysis

The sample did not comply with normality by Shapiro-Wilk, so non-parametric tests were performed. We determined the demographic and anthropometric parameters as means and standard deviations for continuous variables (age, body mass, height, body mass index, Global Deterioration Scale, Four-Meter Gait Speed, Standardized Frailty Criteria, Short Physical Performance Battery, Timed Up and Go Test, Short Falls Efficacy Scale-International and gait parameters of G-STRIDE device) or percentages for the discrete variable (sex, type of surface and level of physical activity). We performed group comparison between the derivation and mean scores with the Mann–Whitney U statistic. Also, the effect size was calculated with Cohen’s statistic and its transformation to correlation coefficient (dr). The following values were considered with respect to the magnitude of the effect size: dr = 0.10 (low), dr = 0.30 (medium), dr = 0.50 (high) and dr = 0.70 (very high) [38]. We demonstrated the concurrent validity of each model based on the intraclass correlation coefficients (ICCs) calculated using a two-way mixed model (absolute agreement type) by comparing the walking speeds estimated from conventional measurement (Four-Meter Gait Speed) and those obtained from G-Stride. Based on the 95 percent confident interval of the ICC estimate, it is often considered very good if ICC is greater than 0.90, good if it is between 0.71 and 0.90, moderate between 0.51 and 0.70, mediocre between 0.31 and 0.50, and bad or null if ICC is less than 0.31. Correlations between variables were analyzed using Spearman test. The analysis of the variables was carried out with the statistical program IBM SPSS Statistics for Windows, version 27.0 (IBM SPSS Corp., Armonk, NY, USA), considering the significant *p* < 0.05.

## 3. Results

### 3.1. Basal Characteristics

Table 1 shows basal characteristics of the study with 21 participants (11 of them fallers). Mean age was 81.1 ± 4.8 years, being older the group of fallers and 12 (57.1%) were women. Regarding physical activity, 45.5% of fallers were sedentary, while 100% of non-fallers were active. In addition, cognitive impairment was only identified in fallers (measured by Reisberg Global Deterioration Scale (GDS).

### 3.2. Correlations between Clinical Tests and Gait Characteristics Obtained by G-Stride Device

**Figure 7:**
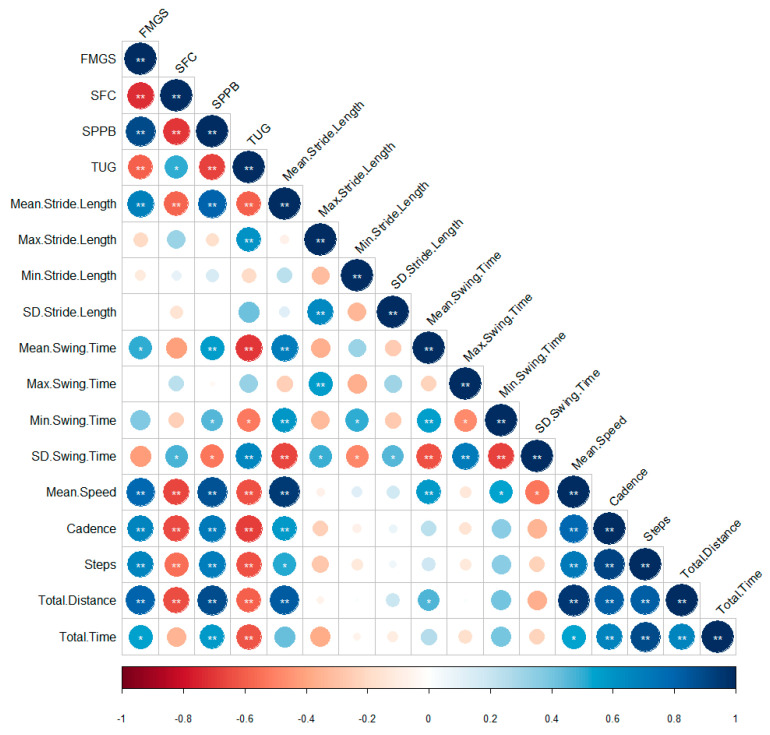
Correlations between Clinical Tests and Gait Characteristics obtained by G-STRIDE Device. The bigger and the more intense the color is, the higher the correlation. Blue means positive correlations and red negative correlations. ** Shows a significant correlation at the 0.01 level and * at the 0.05 level. Four-Meter Gait Speed (FMGS), Standardized Frailty Criteria (SFC), Short Physical Performance Battery (SPPB), Timed Up and Go Test (TUG).

Intraclass correlation coefficient was 0.69 with a 95% confident interval, which indicates moderate reliability of G-Stride device. Figure 7 shows correlations between gait parameters processed by the G-STRIDE sampling device and functional tests commonly used in clinical practice. Mean speed, mean stride, and cadence variables obtained by the device show correlations larger than 0.57 (*p* < 0.05) with functional tests, being the best correlation between mean speed and SPPB (0.87; *p* < 0.01). Also mean swing time shows good correlation with gait speed in 4 m, SPPB, and TUG with correlations between 0.51 and 0.75 (*p* < 0.01), allowing us to analyze the gait pattern with this variable.

### 3.3. Differences in Gait Characteristics between Groups

**Figure 8:**
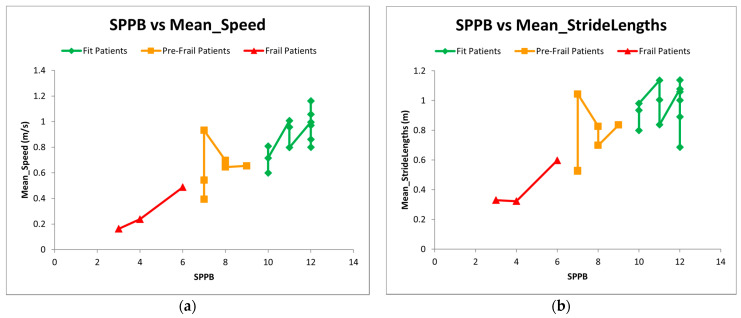
(**a**,**b**) SPPB versus mean Speed and mean Stride Length. Differences in mean speed and mean stride length between frailty groups according to SPPB classification. Each point represents the score in SPPB versus mean speed and mean stride length of each subject.

Differences between fallers and non-fallers are shown in Table 2 for functional tests and Table 3 for G-STRIDE parameters. In comparison to non-fallers, the fallers had worst performance in all functional tests used in clinical practice with significant differences (*p* < 0.000) in all except Short FES-I test. Regarding gait analysis measured by the device, we found significant differences in mean stride length, mean swing time, mean speed, cadence, number of steps, and total distance covered. Additionally, we classify participants according to frailty status using SPPB cut-offs: SPPB < 4 (with disability), SPPB 4–6 (frail), SPPB 7–9 (pre-frail), SPPB 10–12 (non-frail). There were 3 participants in frailty group, 6 in pre-frail group and 12 were non-frail. We found differences in two gait parameters (mean speed and mean stride length) between these frailty groups (Figure 8).

### 3.4. Acceptability of G-STRIDE Device

It is a small and light device (78.71 g); easy and familiar to wear and no problems arose using G-STRIDE device during the 30 min walking test. Data transfer rate through WIFI has been tested, the results show that each minute of data recording requires one minute to send this data to the server by WIFI.

## 4. Discussion

This pilot study aims to describe the development of an inertial INS-based device (the G-STRIDE device) that allows gait assessment without needing any learning or calibration phase and a mobile application (G-STRIDE App) for managing the information to make it accessible to clinicians. The objectives of the study include evaluation of older adults with and without falls using classic functional procedures and inertial device parameters (the G-STRIDE Assessment), and finally, we describe the relationship between gait parameters and frailty status. The study results identify the G-STRIDE device as a reliable and valid tool because it accurately detects the walking risk of falls in older adults and a remarkable concurrent validity compared to classical functional assessment in the clinical setting. G-STRIDE is a novel approach with high accuracy and potential for a larger range of gait parameter estimations (stride length, speed, cadence) when compared with model-based algorithms.

### 4.1. The Algorithm

The algorithms developed for estimating population gait parameters in the G-STRIDE solution showed substantially higher accuracy compared to models based on human gait and abstract regression, which require calibration or training, respectively. This improved accuracy in G-STRIDE was recently verified, using a stride length ground truth reference, in a pilot study of a validation dataset of a small sample size (<3% mean absolute error- MAE in stride length estimation [39]). In line with our study, Byun et al. [23] published a related study using an inertial measurement unit attached to the lower back to measure gait speed in the elderly. The learning phrase showed good results in gait speed estimation, at low speed (4.7% MAE), using a repression estimation method. Likewise, Vallabhajosula et al. [24] conducted a study to validate their technological device based on pressure sensors to measure gait patterns and found that concurrent validity was acceptable. Also, a recent study conducted by Amitrano et al. [40] validates a new wearable e-textile based system (SWEET Sock) for remote gait and postural assessment. It is of utmost importance to incorporate these devices in consultation to speed up the assessment process and to be able to anticipate risks of falls or deterioration of muscle health.

The G-STRIDE solution also can estimate the total travelled distance (TTD), a parameter quite relevant and significant. This parameter is considered of interest as it is correlated with the exhaustion level of a person while walking, especially in elderly [29,41]. The total travelled distance in a test has relevance at large runs of at least 30 min [28], something that cannot be done in clinical spaces or under a direct doctor consultation. Accuracy in TTD for foot-mounted INS-based estimations has been reported to be below 1.5% by previous studies [13,25,27].

### 4.2. The Patients

Based on our results we suggest that older adults without falls had a high level of physical activity, whereas almost 50% of fallers were sedentary. This suggests that gait pattern has a strong relationship with the physical condition and muscle status. Therefore, when identifying people at potential risk of falling, one aspect to focus on is exercise and physical activity to improve muscle mass and muscle performance [8]. In this sense, Jagos et al. [42] found that patients with neurological conditions provided more detectable strides than the control group, so patients had to perform more strides to cross the same distance than healthy subjects. This may increase fatigue, muscle exhaustion and thus the risk of falls.

We found that the group of fallers were older, as described before [37]. Aging is associated with many fall risk factors and significantly linked to frailty and sarcopenia that have consequences as falls, functional decline, and functional disability [43].

Cognitive functions, mainly executive functions, are related to gait pattern, coordination, and balance. They can explain motor syndrome associated with mild cognitive impairment and dementia as described by other authors [40,44,45]. Recently Mulas et al. [46] published the results of gait assessment in Italian cognitive impaired older adults using wearable inertial sensors finding a significant reduction of speed in these patients and other changes in gait parameters.

Regarding functional parameters we found differences between adults with and without falls as it is described in literature [33] but interestingly there are differences in G-STRIDE parameters that allows the device to detect those patients at high risk of falls.

There is good correlation between functional tests and device parameters. Some other authors have compared clinical tests with accelerometry, although they use only TUG to compare with [47].

### 4.3. Pre-Frailty and Frailty Criteria

Additionally, we have found differences in G-STRIDE parameters according to frailty status, which means it is possible to identify patients with pre-frailty and frailty criteria by using the G-STRIDE device. Therefore, clinicians can intervene in order to prevent falls.

A frailty prevalence study [48] observed differences in the pattern and variability of all elderly walking. Frail individuals had a more significant number of shorter walking sessions and less variable in walking bout duration than non-frail older adults. All these features may be restricted to activities entailing short walking bouts (i.e., within the home environment), and they may be unable to sustain prolonged bouts of walking [48]. Furthermore, as Ternero-Quiñones et al. [49] point out, frailty and the risk of falls are significant predictors of autonomy in basic daily life activities.

Falls are a problem of enormous magnitude in the elderly population. Fast, reliable, easy to use, and inexpensive instruments are needed to detect a risk of falls in older adults. Our developed device facilitates assessing gait characteristics in the elderly population with falls, increasing the number of parameters studied, the reliability of the results, and improving the assessment in patients with cognitive impairment who do not collaborate. Also, it reduces consultation times and avoids exposing patients to the hospital environment, which is so important in pandemics.

The main contribution is the development of the algorithm from a pilot sample of clinically older adults and the verification of the accuracy of the algorithm by applying it in a validation set. Another strength of this study is developing an inexpensive, easy-to-use device with WIFI, Bluetooth and microSD. However, there are certain limitations: the sample size is small, so it is necessary to confirm results with a more significant sample. We did not consider the effect of sex on the results: there are differences between groups (54.5% females in fallers and 60% in non-fallers). All non-fallers recruited in the study were physically active and fit, so we could probably consider studying some older adults with no falls less physically active to improve the external validity of our findings.

Additionally, it would be very interesting to confirm results in different pathologies (mild cognitive impairment, dementia, or other neurological disorders) and different populations as pediatrics and to study the utility of the G-STRIDE device to develop personalized treatments and training programs according to patients some main gait parameters.

## 5. Conclusions

Based on the present study’s findings, the G-STRIDE solution appears to be a suitable tool to measure gait parameters in older adults with falls. It may replace conventional tests used in clinical practice because it can efficiently speed up the assessment time, analyzing walking behavior on different surfaces. The device is lightweight and comfortable to wear. Further studies with a larger sample size are needed to be able to draw definitive conclusions.

## Figures and Tables

**Table 1:** Basal Characteristics of Participants.

	Total (*n* = 21)	Non–Fallers (*n* = 10)	Fallers (*n* = 11)	*p* Value
**Age (years)**	81.1 ± 4.8 (73–91)	78.2 ± 3 (73–83)	83.7 ± 4.6 (77–91)	0.011
**Sex (female/male)**	12 (57.1%)/9 (42.9%)	6 (60)/4 (40)	6 (54.5)/5 (45.5)	0.801
**Surface (flat/sloped)**	18 (85.7%)/3 (14.3%)	7 (70%)/3 (30%)	11 (100%)/0 (0%)	0.050
**Physical Activity (No/Yes)**	16 (76.2%)/5 (23.8%)	10 (100%)/0 (0%)	6 (54.5%)/5 (45.5%)	0.015
**Body Mass (kg)**	63.3 ± 9.6 (45–66)	66.8 ± 8.9 (54–86)	60.1 ± 9.8 (46–79)	0.398
**Height (m)**	1.61 ± 0,08 (1.40–1.79)	1.64 ± 0.05 (1.60–1.72)	1.56 ± 0.10 (1.40–1.79)	0.091
**BMI (Kg/m^2^)**	24.5 ± 2.5 (19.9–31.6)	24.6 ± 3.3 (21.1–31.6)	24.4 ± 1.8 (19.9–26.4)	0.015
**GDS**	2.1 ± 1 (1–6)	1 ± 0 (1–1)	3.20 ± 1.8 (1–6)	0.001

Age, Body Mass, Body Height, Body Mass Index (BMI) and Global Deterioration Scale (GDS) are presented with mean ± standard deviation values and range. Sex, Surface and Physical Activity are presented with the points score and percentage.

**Table 2:** Differences between groups functional tests.

Functional Tests	Fallers (Mean ± SD)	Non-Fallers (Mean ± SD)	Sig.	Z	dr
**FMGS (m/s)**	1.13 ± 0.70	1.92 ± 0.34	0.002	−3.02	0.58
**SFC**	1.55 ± 1.37	0.1 ± 0.32	0.004	−2.90	0.57
**SPPB**	7.36 ± 2.54	11.30 ± 0.82	0.001	−3.33	0.72
**TUG (s)**	21.85 ± 24.59	7.72 ± 1.52	0.000	−3.80	0.37
**Short FES-I**	12.64 ± 5.10	9.00 ± 2.49	0.080	−1.75	0.41

Results of the tests: Four-Meter Gait Speed (FMGS), Standardized Frailty Criteria (SFC), Short Physical Performance Battery (SPPB), Timed Up and Go Test (TUG), Short Falls Efficacy Scale-International (Short FES-I). Standard deviation (SD).

**Table 3:** Differences between groups G-STRIDE parameters.

G-STRIDE Parameters	Fallers (Mean ± SD)	Non-Fallers (Mean ± SD)	Sig.	Z	dr
**Mean Stride Length (m)**	0.68 ± 0.24	0.97 ± 0.15	0.007	−2.68	0.58
**SD Stride Length (m^2^)**	0.08 ± 0.06	0.108 ± 0.04	0.180	−1.34	0.28
**Mean Swing Time (s)**	0.75 ± 0.11	0.83 ± 0.06	0.053	−1.94	0.45
**SD Swing Time (s^2^)**	0.04 ± 0.04	0.02 ± 0.03	0.460	−0.74	0.19
**Mean Speed (m/s)**	0.58 ± 0.25	0.91 ± 0.17	0.005	−2.82	0.60
**Cadence (steps/min)**	46.63 ± 8.25	53.11 ± 6.13	0.035	−2.11	0.40
**Steps**	1267.27 ± 415.44	1637.70 ± 278.62	0.041	−2.04	0.46
**Total Distance (m)**	905.56 ± 459.42	1650.09 ± 374.02	0.001	−3.31	0.66
**Total Walking Time (s)**	1593.96 ± 347.44	1819.49 ± 138.64	0.105	−1.62	0.39

Results of G-STRIDE parameters. Standard deviation (SD).

## Supplementary Materials

The following is available online at https://www.mdpi.com/article/10.3390/s21134334/s1, Video S1: G-Stride video.
